# Accuracy of a Real-Time, Computerized, Binocular, Three-Dimensional Trajectory-Tracking Device for Recording Functional Mandibular Movements

**DOI:** 10.1371/journal.pone.0163934

**Published:** 2016-10-04

**Authors:** Tian Zhao, Huifang Yang, Huaxin Sui, Satyajeet Sudhir Salvi, Yong Wang, Yuchun Sun

**Affiliations:** 1 Center of Digital Dentistry, Peking University School and Hospital of Stomatology, Beijing, China; 2 Faculty of Prosthodontics, Peking University School and Hospital of Stomatology, Beijing, China; 3 National Engineering Laboratory for Digital and Material Technology of Stomatology, Beijing, China; 4 Research Center of Engineering and Technology for Digital Dentistry, Ministry of Health, Beijing, China; 5 Beijing Key Laboratory of Digital Stomatology, Beijing, China; Duke University, UNITED STATES

## Abstract

**Objective:**

Developments in digital technology have permitted researchers to study mandibular movements. Here, the accuracy of a real-time, computerized, binocular, three-dimensional (3D) trajectory-tracking device for recording functional mandibular movements was evaluated.

**Methods:**

An occlusal splint without the occlusal region was created based on a plaster cast of the lower dentition. The splint was rigidly connected with a target on its labial side and seated on the cast. The cast was then rigidly attached to the stage of a high-precision triaxial electronic translator, which was used to move the target-cast-stage complex. Half-circular movements (5.00-mm radius) in three planes (XOY, XOZ, YOZ) and linear movements along the x-axis were performed at 5.00 mm/s. All trajectory points were recorded with the binocular 3D trajectory-tracking device and fitted to arcs or lines, respectively, with the Imageware software. To analyze the accuracy of the trajectory-tracking device, the mean distances between the trajectory points and the fitted arcs or lines were measured, and the mean differences between the lengths of the fitted arcs’ radii and a set value (5.00 mm) were then calculated. A one-way analysis of variance was used to evaluate the spatial consistency of the recording accuracy in three different planes.

**Results:**

The mean distances between the trajectory points and fitted arcs or lines were 0.076 ± 0.033 mm or 0.089 ± 0.014 mm. The mean difference between the lengths of the fitted arcs’ radii and the set value (5.00 mm) was 0.025 ± 0.071 mm. A one-way ANOVA showed that the recording errors in three different planes were not statistically significant.

**Conclusion:**

These results suggest that the device can record certain movements at 5.00 mm/s, which is similar to the speed of functional mandibular movements. In addition, the recordings had an error of <0.1 mm and good spatial consistency. Thus, the device meets some of the requirements necessary for recording human mandibular movements.

## Introduction

The human mandible is an articulated bony structure that moves significantly during activities such as chewing, swallowing, and speaking. Research on such functional mandibular movements is complex and challenging, as key parameters such as the incisal and condylar guidance inclinations, two-dimensional (2D) measurements of border movements, and three-dimensional (3D) trajectory data must be accurately acquired [1]. Thanks to the development of new measurement and analysis techniques, 3D trajectory data can now be saved in text format and imported into virtual articular software to recreate individual mandibular movements. Information obtained from examining these movements can be used clinically to diagnose temporomandibular and masticatory muscle disorders and to help design functional occlusal morphology for dental restorations [2].

Traditionally, approximate mandibular movements are simulated by adjustable mechanical articulators. Cone-beam computed tomography (CT), dynamically enhanced magnetic resonance imaging, and trajectory tracking have been used to acquire individual mandibular trajectories [3], with the trajectory tracking technique being the most popular method. In these devices, the maxillary part is usually fixed to the bilateral external auditory canals and nasal bridge; however, since these structures are not rigid, uncontrollable errors can be generated by the relative motion of the maxillary region during mandibular movements. Further, the weight of the mandible itself can interfere with mandibular movements. Unfortunately, ensuring that the entire device can be appropriately attached to a human head with minimal weight is very complicated and expensive.

To overcome these issues, a real-time, computerized, binocular, 3D trajectory-tracking device (Peking University School and Hospital of Stomatology, China) was developed with a calibrated accuracy of approximately ±0.1 mm [4]. This device was designed to record typical border movements including protrusive, lateral, and opening movements, as well as habitual movements of the mandible during functions such as chewing and swallowing. The recording accuracy of the device was also evaluated in this study.

## Methods

### Ethics statement

This study, including the procedures for obtaining the plaster cast, was approved by the Bioethics Committee of Peking University School and Hospital of Stomatology, Beijing, China (No. PKUSSIRB-201412008; Date: 5/19/2014). In this study, the plaster cast we used was from a single human experimental subject. Written informed consent to publish these case details was obtained from this individual.

### Cast and occlusal splint preparation

One patient (female, 25 years old) who was admitted to the Department of Prosthodontics at Peking University School and Hospital of Stomatology was enrolled in this study after providing written informed consent. In accordance with the requirements outlined in a prosthodontics textbook [5], a mandibular impression of the patient was obtained, and then plaster was poured into a standard cast base to prepare the plaster cast of the lower dentition. An occlusal splint was fabricated based on the plaster cast using 1-mm thick thermoplastic material (Erkodent Erich Kopp GmbH, Pfalzgrafenweiler, Germany), and its occlusal part was removed to prevent occlusion interference.

### Setup of the experimental platform

**Fig 1:**
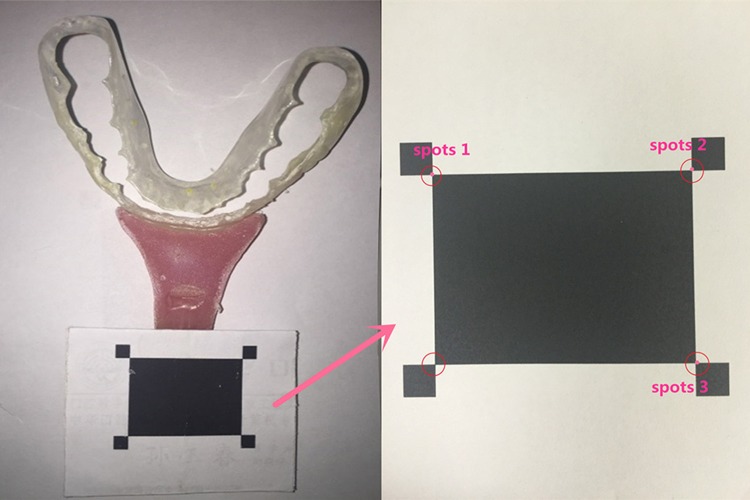
The occlusal splint was rigidly attached to a target. The trajectories of three spots within the target were recorded using the computerized, binocular, 3D trajectory-tracking device.

**Fig 2:**
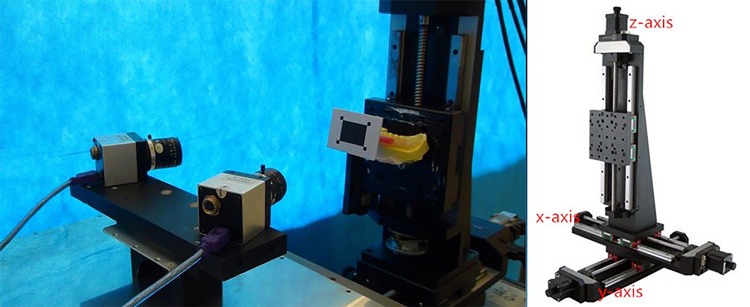
The plaster cast assembly was rigidly fixed to the stage of a high-precision triaxial electronic translator. The translation stage could move linearly along the x-, y-, and z-axes, as well as circularly in three planes (XOY, XOZ, and YOZ) with a recording accuracy of 0.001 mm.

A high-precision, three-axis, electronic translator (Beijing stand upright, Han Optical Instrument Co. Ltd, China, Beijing) was employed. In the coordinate system of the translator, its translation stage could be moved linearly along the x-, y-, and z-axes, as well as circularly in three coordinate planes (XOY, XOZ, and YOZ), to perform given movements (Fig 1) with an accuracy of 0.001 mm. Light-curing resin material (Megatray Base Plate, Megadenta Dental Products GmbH, Radeberg, Germany) was used to rigidly fix a specialized target to the labial region of the occlusal splint (Fig 1); then, the plaster cast was rigidly attached to the translation stage (Fig 2). The target consisted of a central black rectangle (32 × 23.5 mm) and four corner black squares (4 × 4 mm). Three spots on the target were selected for tracking the trajectories (Fig 1). The stage was centered (point O) and positioned to ensure all the given movements to be within the effective measuring area of the trajectory-tracking device [4].

### Half-circular trajectory recordings and the related accuracy and spatial consistency evaluations

**Fig 3:**
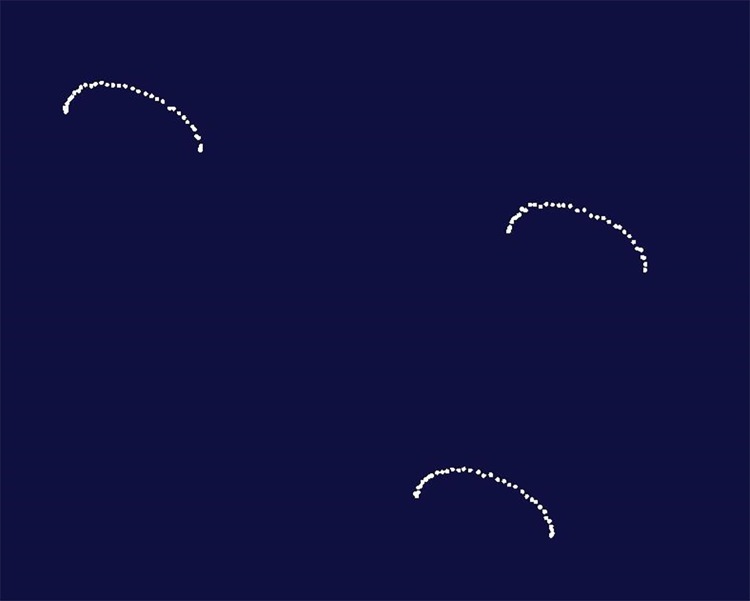
The curved (arc) trajectories of the three target spots in one plane were shown in white.

The translation stage was set in half-circular movement with a 5.00-mm radius at 5.00 mm/s in three coordinate planes (XOY, XOZ, YOZ), respectively, and the trajectories of the three target spots were recorded in real time; these movements and measurements were repeated five times (Fig 3). The recorded data is a series of coordinate points reflecting the trend of performed trajectories. Then the trajectory points were imported to the Imageware 13.2 software (Siemens PLM Software, Plano, Germany) (Fig 3) and fitted to arcs by approximating specific clouds to find the best fit with the “arc creation” tool. The lengths of the radii and the distance between each tracked trajectory point and the fitted arcs were measured with the “cloud deviation” tool; and the mean values were calculated to determine the accuracy. A one-way analysis of variance was used to evaluate the spatial consistency of the recording accuracy in three different planes.

### Linear trajectory recordings and the related accuracy evaluations

**Fig 4:**
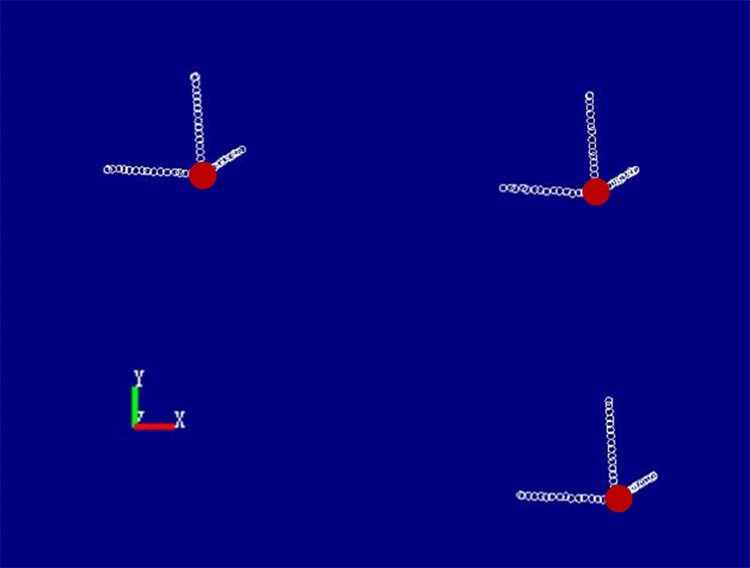
The initial positions of the three target spots were shown in red. The linear trajectories of the three target spots in three directions were shown in white.

To determine the accuracy of the device when recording linear movements, the translation stage was set to move at 5.00 mm/s, with a movement time of 1 s, along the x-axis; the movement and recording was repeated five times. The trajectory points were imported to Imageware (Fig 4) and fitted to straight lines with the “straight-line creation” tool. Finally, the mean distances between the tracked trajectory points and the fitted lines for the three target spots were measured with the above-mentioned method.

## Results

Collectively, the mean difference between the length of the fitted arcs’ radii and the set value (5.00 mm) was 0.025 ± 0.071 mm; the mean distance between the trajectory points and the fitted arcs was 0.076 ± 0.033 mm (Table 1).

In the XOY, XOZ, and YOZ planes, the mean differences between the lengths of the fitted arcs’ radii and the set value (5.00 mm) were 0.001 ± 0.045 mm, 0.001 ± 0.039 mm, and 0.073 ± 0.093 mm, respectively. The P value of the one-way analysis of variance among the three planes is 0.948. The mean distances between the trajectory points and the fitted arcs for the three planes were 0.065 ± 0.021 mm, 0.094 ± 0.031 mm, and 0.068 ± 0.039 mm, respectively. The P value of the one-way analysis of variance among the three planes is 1.000. The one-way analysis of variance showed that the recording errors in the three different planes were not statistically significant (P >0.05).

The mean distance between the trajectory points and fitted lines was 0.089 ± 0.014 mm.

**Table 1:** Recording errors of the device.

Evaluation index	Evaluation content	D-value	Mean value
**Recording error for the curve (arc) trajectory from thethree target spots, collectively**	The mean difference between the radii of the fitted arcs and the set value		0.032 ± 0.071
	The mean distance from each tracked trajectory point in the half-circular movement to the fitted arcs		0.076 ± 0.033
**Spatial consistency of the measurements from the three planes, respectively**	The mean difference between the radii of the fitted arcs for the half-circular movement and the set values	0.001	0.025 ± 0.071
		0.001	
		0.073	
	The distance from each tracked trajectory point in the half-circular movement to the fitted arcs	0.065	0.076 ± 0.033
		0.094	
		0.068	
**Recording error for the linear trajectory**	The distance from each tracked trajectory point during the linear movement to the fitted lines of the three target areas in one direction		0.089 ± 0.014

D-value: difference between the observed value and the pre-set value.

## Discussion

By recording and examining the individual mandibular movement trajectory, important basic information about the movements can be collected, and such information may be useful for designing functional occlusions. Different restorations require different accuracies, and restorations supported by implantations require the highest level of accuracy. In this study, our objective was to evaluate the accuracy of a real-time, computerized, binocular, 3D trajectory-tracking device to determine its utility as an innovative tool for dental applications.

Mandibular movement is complicated, but it can be divided into some basic physical parts such as translational and rotational, or to say geometric parts such as rectilinear and circular motions. The present study utilized a high-precision three-axis electronic translator to perform these basic movements. By recording the basic movements with the computerized, binocular, 3D trajectory-tracking device and comparing them to a set value, we were able to calculate the recording accuracy of the device.

Collectively, the results revealed that the recording error was <0.1 mm. When measuring the lengths of the radii in each fitted arc, the largest error was found in the YOZ plane (~0.1 mm), while the errors in the other two planes were <0.001 mm. Since the movement of the electronic translation stage was sufficiently accurate (0.001 mm), and since the entire target-cast-stage complex was fixed to generate a single rigid entity, the observed errors are most likely related to the performance of the device itself. To a certain extent, the curved (arc) and linear trajectories in the three different coordinate planes reflect the 3D aspect of mandibular movement. Furthermore, an effective measuring area was set within the trajectory-tracking device to ensure that all of the mandibular border movements were recorded within this area.

Compared with other technologies mentioned in the introduction section, the trajectory-tracking device we developed had three major advantages. First, when recording human mandibular movements, the part that was fixed on the head (harder tissue) was more stable [4]. Second, the target that was connected to the lower dentition was light, thus there was less interference with the mandibular movements. Finally, the entire device was supported by the ground, thus a less expensive, larger, and simpler binocular visual system can be applied; on no case that all parts of the device were loaded on the subject.

The present study primarily quantitatively evaluated the accuracy of the computerized, binocular, mandibular 3D trajectory-tracking device itself. However, the main limitation of this device is that slight head movements can reduce the accuracy when recording mandibular movements. Further research should consider the effects of head movements by applying a head-fixing frame to the device.

## Conclusion

Our results suggest that the real-time, computerized, binocular, 3D trajectory-tracking device can record given movements at 5.00 mm/s, which is similar to the speed of functional mandibular movements. Importantly, the error was <0.1 mm and the device had good spatial consistency. The accuracy of this device indicates that it meets some of the requirements necessary for recording human mandibular movements.

## Supporting Information

S1 Table Recording errors of the device. This table cantains a summarized table and detailed data of recording errors.
(XLSX)
